# Mice that lack the C-terminal region of Reelin exhibit behavioral abnormalities related to neuropsychiatric disorders

**DOI:** 10.1038/srep28636

**Published:** 2016-06-27

**Authors:** Kaori Sakai, Hirotaka Shoji, Takao Kohno, Tsuyoshi Miyakawa, Mitsuharu Hattori

**Affiliations:** 1 Department of Biomedical Science, Graduate School of Pharmaceutical Sciences, Nagoya City University, Nagoya, Aichi, Japan; 2 Division of Systems Medical Science, Institute for Comprehensive Medical Science, Fujita Health University, Toyoake, Aichi, Japan; 3 Section of Behavior Patterns, Center for Genetic Analysis of Behavior, National Institute for Physiological Sciences, Okazaki, Aichi, Japan

## Abstract

The secreted glycoprotein Reelin is believed to play critical roles in the pathogenesis of several neuropsychiatric disorders. The highly basic C-terminal region (CTR) of Reelin is necessary for efficient activation of its downstream signaling, and the brain structure of knock-in mice that lack the CTR (ΔC-KI mice) is impaired. Here, we performed a comprehensive behavioral test battery on ΔC-KI mice, in order to evaluate the effects of partial loss-of-function of Reelin on brain functions. The ΔC-KI mice were hyperactive and exhibited reduced anxiety-like and social behaviors. The working memory in ΔC-KI mice was impaired in a T-maze test. There was little difference in spatial reference memory, depression-like behavior, prepulse inhibition, or fear memory between ΔC-KI and wild-type mice. These results suggest that CTR-dependent Reelin functions are required for some specific normal brain functions and that ΔC-KI mice recapitulate some aspects of neuropsychiatric disorders, such as schizophrenia, bipolar disorder, and autism spectrum disorder.

Reelin, originally identified as a protein that is deficient in an autosomal recessive mutant mouse strain *reeler*, is a secreted glycoprotein that is essential for the organization of brain structures^1^. In mature brain, Reelin is expressed mainly in interneurons in the cerebral cortex and hippocampus^2^ and modulates neuronal function and synaptic plasticity^3^. Reelin binds to very low-density lipoprotein receptor (VLDLR) and apolipoprotein E receptor 2 (ApoER2) and induces phosphorylation of the intracellular protein Dab1, which leads to activation or modulation of further downstream molecules^4^. Phosphorylated Dab1 is quickly degraded by the ubiquitin-proteasome pathway^5^. The intracellular Reelin signaling pathway regulates changes in cytoskeletal organization^6^, protein distribution^7^, cell polarity^8^, and gene expression^9^. Not surprisingly, even a slight change in Reelin activity would greatly affect brain structure and function. In humans, disturbance or hypoactivity in Reelin signaling has been suggested to play a role in several neurodevelopmental psychiatric disorders^3^,^10^, including schizophrenia, bipolar disorders, and autism spectrum disorders (ASD). For these reasons, validating an animal model in which Reelin activity is dysregulated is significant not only for understanding of the pathophysiology of neuropsychiatric disorders but also for the development of novel therapeutics.

Heterozygous *reeler* mice (HRM) that express approximately half the wild-type (WT) amount of Reelin have been proposed as an animal model of neurodevelopmental psychiatric disorders^11^,^12^,^13^, because they have been reported to have behavioral deficits such as abnormal prepulse inhibition (PPI), motor impulsivity, motor stereotypies, and contextual fear conditioning. In rats, prenatal restraint stress decreased Reelin expression in the brain, possibly by affecting the DNA methylation levels of the Reelin promoter, and induced excessive spontaneous locomotor activity, increased anxiety-like behavior, and learning and memory deficits. These data support the idea that downregulation of Reelin signaling by prenatal stress is a strong risk for schizophrenia and bipolar disorders^14^. Finally, injection of Reelin into the ventricles^15^ or genetic Reelin overexpression^16^ rescue the cognitive impairment of HRM, indicating that upregulation of Reelin function could ameliorate some aspects of related neuropsychiatric disorders.

However, using HRM as a model of human disorders has not achieved a wide consensus. Qiu *et al*.^17^ reported that HRM show normal overall activity, coordination, startle responses, anxiety-like behavior, electronic shock sensitivity, cued freezing behavior, and spatial learning, although they exhibited a significant reduction in contextual fear conditioned learning. Podhorna and Didriksen^18^ argued that HRM exhibit normal behavior in a wide range of behavioral measures. In fact, two very recent studies have shown that the behavioral phenotypes of HRM are fairly marginal and can be influenced by many other factors^19^,^20^. Finally, adult-specific knock-out (KO) of Reelin has only mild effects on the behaviors^21^. Because of this controversy, additional or alternative model in which Reelin functions are impaired is required if one is to investigate the causal relationship between Reelin and neuropsychiatric disorders. It is also imperative to test partial loss-of-function of Reelin because Reelin appears to have multiple functions in the normal brain function.

We recently generated knock-in mice in which the positively-charged C-terminal region (CTR) of Reelin is deleted (ΔC-KI mice)^22^. CTR is required for efficient activation of the downstream signaling pathway^23^; therefore Reelin signaling is attenuated in ΔC-KI mice^22^. Although neuronal migration during embryonic development is normal in ΔC-KI mice, there are some abnormalities in the structure of the cerebral cortex and hippocampus in early postnatal stages of ΔC-KI mice development^22^. In this study, we analyzed the behavior of ΔC-KI mice via a comprehensive behavioral test battery. The results indicate important roles of Reelin in higher brain functions and the potential of ΔC-KI mice as a validated model of neurodevelopmental psychiatric disorders.

## Results

### Brain structure of the adult ΔC-KI mice was partially disturbed and Reelin signaling of them was attenuated in the cerebral cortex and hippocampus

**Figure 1:**
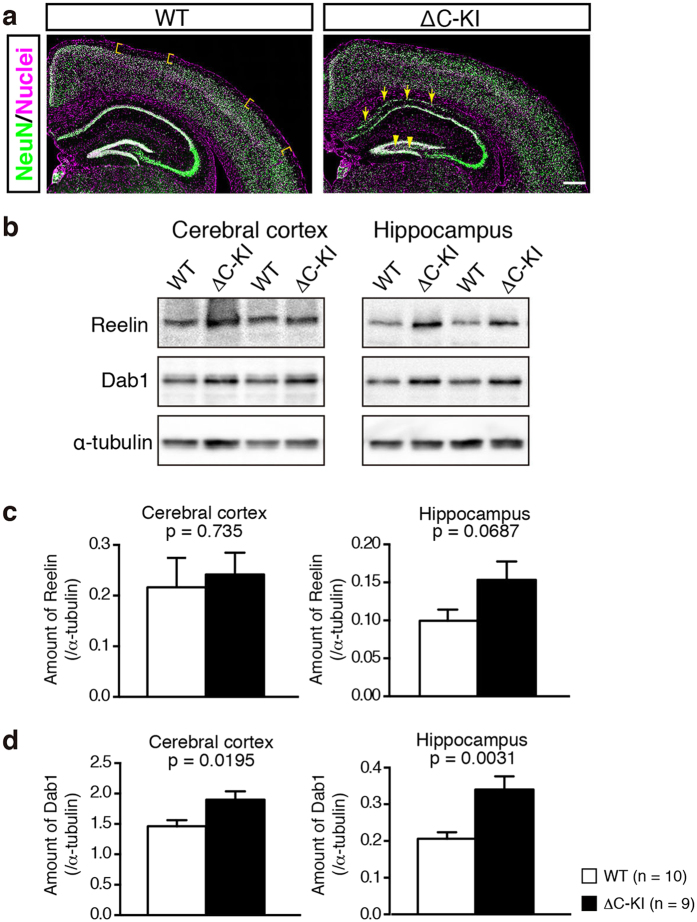
Structural and biochemical phenotypes of adult ΔC-KI mice. (**a**) Adult brain sections of wild-type (WT) (left) or ΔC-KI (right) mice were immunostained with anti-NeuN (green). The nuclei were stained with Hoechst 33342 (magenta). Scale bar, 400 μm. Yellow U shapes in the left panel indicate the marginal zone (MZ), which is blurred in ΔC-KI mice. Yellow arrows in the right panel indicate mislocalized neurons in the hippocampus of ΔC-KI mice. The yellow arrowhead in the right panel indicates the less-packed dentate gyrus of ΔC-KI mice. (**b**) The homogenates of adult cerebral cortex or hippocampus from WT and ΔC-KI mice were analyzed by western blotting. (**c,d**) Quantification of the amount of Reelin (**c**) and Dab1 (**d**) in the cerebral cortex and hippocampus. Bars represent mean ± SEM. Unpaired t-tests were used to test for statistical significance.

In our previous analysis of neonatal ΔC-KI mice, we found that the marginal zone or layer I of the cerebral cortex was narrower than that of WT mice. In addition, the hippocampal CA1 pyramidal cell layer was split into two layers, and the dentate granule cell layer was less densely packed^22^. Here we confirmed that these structural abnormalities persist into adulthood (Fig. 1a). Western blotting (WB) using adult brain homogenate suggested increased Reelin protein levels in ΔC-KI mice (Fig. 1b, top panels). However, unlike the similar increase seen neonatally^22^, the difference in adults was not statistically significant in any brain area analyzed (Fig. 1c; cerebral cortex, t_17_ = 0.344, p = 0.735; hippocampus, t_17_ = 1.944, p = 0.0687). Dab1 protein in the cerebral cortex or hippocampus was significantly increased in ΔC-KI mice versus WT mice (Fig. 1b, middle panels, and Fig. 1d; cerebral cortex, t_17_ = 2.58, p = 0.0195; hippocampus, t_17_ = 3.448, p = 0.0031). These data indicated that Reelin signaling continues to be attenuated in the adult brains of ΔC-KI mice, as in that of neonatal ones.

### General characteristics of adult ΔC-KI mice

**Figure 2:**
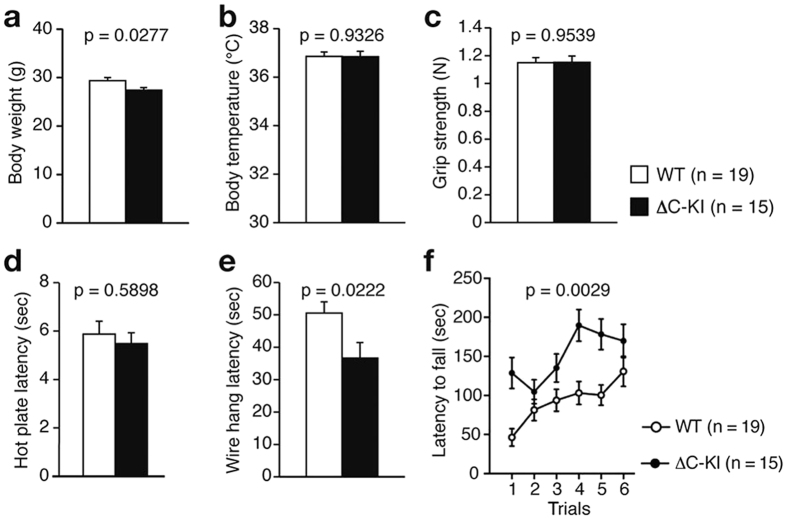
General characteristics of ΔC-KI mice. (**a**) Body weight. (**b**) Body temperature. (**c**) Grip strength. (**d**) Latency to the first fore- or hind-paw response in the hot plate test. (**e**) Wire hang latency. (**f**) Latency to fall off the rod in the rotarod test. Bars represent mean ± SEM. Unpaired t-tests (**a–e**) and a two-way repeated measures ANOVA (**f**) were used to test for statistical significance.

The mean body weight of ΔC-KI mice was lower than that of WT mice (Fig. 2a; t_32_ = 2.31, p = 0.0277), which may be due to their hyperactivity (see below), or to reduced or delayed physical development. However, ΔC-KI mice showed no abnormalities in body temperature (Fig. 2b; t_32_ = 0.09, p = 0.9326), grip strength (Fig. 2c; t_32_ = 0.06, p = 0.9539), or pain sensitivity (Fig. 2d; t_32_ = 0.54, p = 0.5898). ΔC-KI mice fell from a wire lid earlier than WT mice in the wire hang test (Fig. 2e; t_32_ = 2.4, p = 0.0222); however, they remained longer on the rotating wheel in the rotarod test (Fig. 2f; genotype effect, F_1, 32_ = 10.41, p = 0.0029; genotype × trial interaction, F_5, 160_ = 2.68, p = 0.0234).

### ΔC-KI mice were hyperactive and exhibited reduced anxiety-like behavior

**Figure 3:**
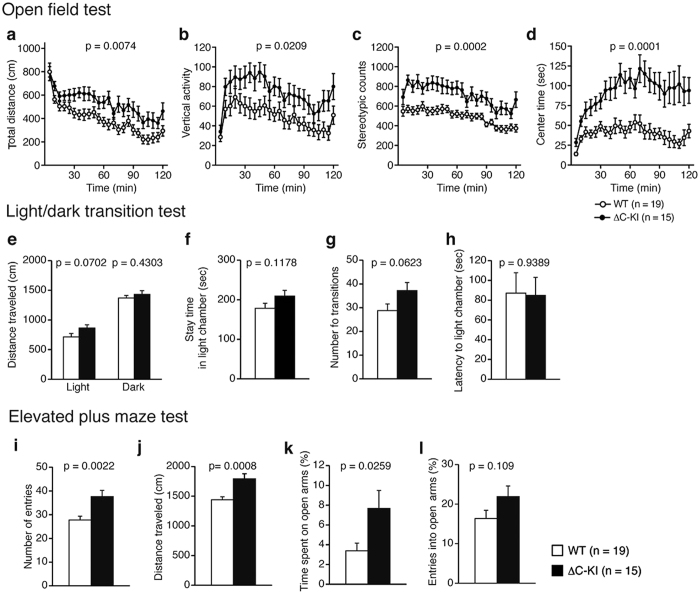
Hyperactivity and reduced anxiety-like behavior of ΔC-KI mice. (**a–d**) Open field test: total distance traveled (**a**), the quantity of vertical activity (**b**), the number of stereotypic counts (**c**), and center time (**d**). (**e–h**) Light/dark transition test: distance traveled (**e**), stay time in light chamber (**f**), the number of transitions (**g**), latency to enter light chamber (**h**). (**i–l**) Elevated plus maze test: the number of entries (**i**), distance traveled (**j**), the percentage of time spent on open arms (**k**), the percentage of entries into open arms (**l**). Bars represent mean ± SEM. Unpaired t-tests (**e–l**) and two-way repeated measures ANOVA (**a–d**) were used to test for statistical significance.

In the open field test, the ΔC-KI moved a longer total distance than WT mice (Fig. 3a; genotype effect, F_1, 32_ = 8.17, p = 0.0074; genotype × time interaction, F_23, 736_ = 1.03, p = 0.426), indicating that the ΔC-KI mice were hyperactive. Vertical activity (Fig. 3b; rearing measured by counting the number of photobeam interruptions, genotype effect, F_1, 32_ = 5.9, p = 0.0209; genotype × time interaction, F_23, 736_ = 0.72, p = 0.8289) and stereotypy counts (Fig. 3c; defined by the number of breaks of the same beam, genotype effect, F_1, 32_ = 17.88, p = 0.0002, genotype × time interaction, F_23, 736_ = 1.18, p = 0.2594) were also significantly increased in ΔC-KI mice. These results are also further indication of hyperactivity in the ΔC-KI mice. Time spent in the center area of the field (center time), which is a measure of anxiety-like behavior (increased center time is considered as reduced anxiety), was markedly increased in ΔC-KI mice (Fig. 3d; genotype effect, F_1, 32_ = 19.61, p = 0.0001, genotype × time interaction, F_23, 736_ = 2.57, p < 0.0001). However, hyperactivity may also contribute to the increased time in the center area in ΔC-KI mice. In the light/dark transition test, there was no difference of any parameter examined between ΔC-KI and WT mice (Fig. 3e–h), suggesting that anxiety-like behavior in this paradigm was unchanged in ΔC-KI mice. Interestingly, in the elevated plus maze test, ΔC-KI mice showed significant increases in the number of entries into arms (Fig. 3i; t_32_ = 3.34, p = 0.0022), distance traveled (Fig. 3j; t_32_ = 3.71, p = 0.0008), and time spent on open arms (Fig. 3k; t_32_ = 2.34, p = 0.0259). The number of entries into open arms tended to increase in ΔC-KI mice, although it was not statistically significant (Fig. 3l; t_32_ = 1.65, p = 0.109). These results indicate that ΔC-KI mice exhibit hyperactivity and reduced anxiety-like behavior in open space and high places.

### ΔC-KI mice showed normal depression-like behavior

There was no difference in the percentage of immobility time between ΔC-KI and WT mice in the Porsolt forced swim test (Supplementary Fig. S1a; Day 1, F_1, 32_ = 1.06, p = 0.3109; Day 2, F_1, 32_ = 2.36, p = 0.1343) or in the tail suspension test (Supplementary Fig. S1b; F_1, 30_ = 0.71, p = 0.405). Therefore, ΔC-KI mice showed normal depression-like behavior.

### Sociability of ΔC-KI mice was partially impaired, whereas social novelty preference was normal

**Figure 4:**
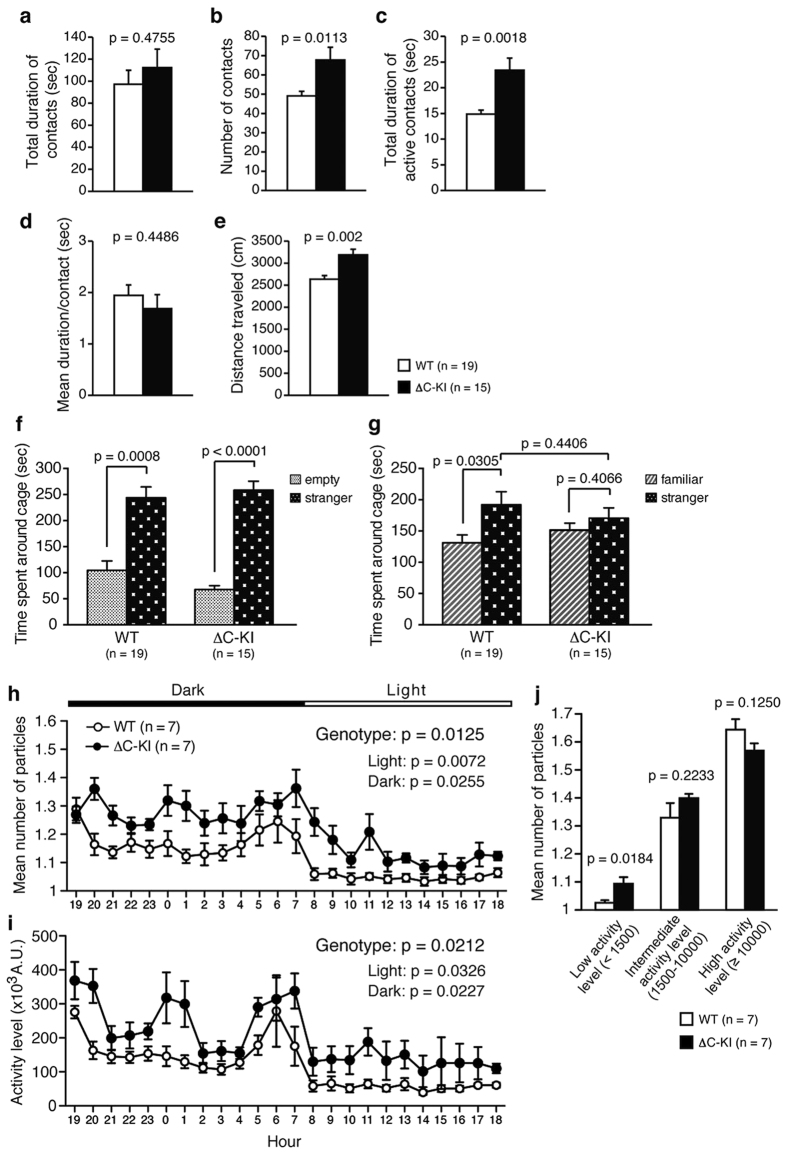
Social behavior of the ΔC-KI mice is partially impaired. (**a–e**) Social interaction test in a novel environment: The total duration of contacts (**a**), the number of contacts (**b**), the total duration of active contacts (**c**), the mean duration per contact (**d**), and the distance traveled (**e**). (**f,g**) Crawley’s three-chamber social approach test: Time spent around cage in the sociability test (**f**), time spent around cage in the social novelty preference test (**g**). (**h–j**) Home cage social interaction test: Mean number of particles (**h**), activity level (**i**) and mean number of particles during low, high, or intermediate activity periods (**j**). Bars represent mean ± SEM. Unpaired t-test (**a–g**,**j**), paired t-test (**f**,**g**), and two-way repeated measures ANOVA (**h,i**) were used to test for statistical significance.

In the social interaction test under novel environment, there was no difference in total duration of contacts (Fig. 4a; t_14_ = 0.73, p = 0.4755) and mean duration of contact (Fig. 4d; t_14_ = 0.78, p = 0.4486). The number of contacts (Fig. 4b; t_14_ = 2.92, p = 0.0113) and total duration of active contacts (Fig. 4c; t_14_ = 3.83, p = 0.0018) were increased in ΔC-KI mice, but these were likely to derive from increased overall activity, as indicated by the concomitant greater distance traveled (Fig. 4e; t_14_ = 3.79, p = 0.002).

We also performed Crawley’s three-chamber social approach test, which consists of sociability and social novelty preference tests. In the sociability test, a wire cage containing a stranger mouse was placed in one side of the chamber and an empty cage was placed on the other side. In the social novelty preference test, a cage of a familiar mouse was placed in one side of the chamber and a cage of a stranger mouse was placed on the other side. Times spent around the wire cage containing the stranger mouse vs. the empty cage, and around the cage containing the familiar mouse vs. the cage with the stranger mouse, are shown in Fig. 4f,g, respectively. In both genotypes, time spent around the cage with a stranger was longer than that with no mouse (Fig. 4f; WT, t_18_ = 4.04, p = 0.0008; ΔC-KI, t_14_ = 8.74, p < 0.0001). WT mice spent more time around the cage with a stranger than around the cage with a familiar mouse, whereas ΔC-KI mice spent comparable time around each cage (Fig. 4g; WT, t_18_ = 2.35, p = 0.0305; ΔC-KI, t_14_ = 0.86, p = 0.4066). However, the total time spent around the cage with a stranger between genotypes was statistically indistinguishable (Fig. 4g; t_32_ = 0.78, p = 0.4406).

To evaluate social interaction in the home cage, two mice of the same genotype that had been housed separately were placed into the cage and were monitored for 1 week. The mean activity level and the mean number of particle detected (two particles are counted when the mice are separated, and one particle is found when they are together) over the last 3 days were calculated. ΔC-KI mice spent more time separated from each other than WT (Fig. 4h; genotype effect, F_1, 12_ = 8.6, p = 0.0125, genotype × time interaction, F_23, 276_ = 1.58, p = 0.0463). Also, the activity level of ΔC-KI mice was increased compared to WT mice throughout the session (Fig. 4i; genotype effect, F_1, 12_ = 7.02, p = 0.0212, genotype × time interaction, F_23, 276_ = 0.96, p = 0.5115). The increased number of particles (i.e., increased separation time) could be the consequence of the increased locomotor activity and thus the increased number of particles in ΔC-KI mice may not reflect a decrease in social interaction itself. Therefore, we evaluated the mean number of particles during immobile, moderately active, or highly active periods (two mice were considered to be immobile when the number of pixels changed was less than 1,500 for a 1-min period; see Materials and Methods for details). The mean number of particles during immobility was significantly greater in ΔC-KI mice than in WT mice, while no genotype difference in the mean number of particles was found during moderately active or highly active periods (Fig. 4j and Supplementary Fig. S2; genotype effect, F_1, 12_ = 0.44, p = 0.5193, genotype × activity level interaction, F_2, 24_ = 5.23, p = 0.013; low activity: p = 0.0184; intermediate activity: p = 0.2233; high activity: p = 0.1259). These results indicated that ΔC-KI mice tend to be separated from each other and suggest that sociability is reduced.

### Acoustic startle response and prepulse inhibition of ΔC-KI mice were normal

There was no difference between ΔC-KI and WT mice in the acoustic startle response at both 110 dB (t_32_ = 0.21, p = 0.8368) and 120 dB (t_32_ = 0.4, p = 0.6895) (Supplementary Fig. S3a); nor were there differences in startle response or prepulse inhibition (PPI) following testing with any combination of prepulse stimulus (74 or 78 dB) and startle stimulus (110 or 120 dB) (Supplementary Fig. S3b; 74 dB prepulse and 110 dB startle, t_32_ = 0.5, p = 0.6192; 78 dB prepulse and 110 dB startle, t_32_ = 0.71, p = 0.4857; 74 dB prepulse and 120 dB startle, t_32_ = 1.95, p = 0.0595; 78 dB prepulse and 120 dB startle, t_32_ = 1.12, p = 0.2714).

### Spatial reference memory in ΔC-KI mice was normal

**Figure 5:**
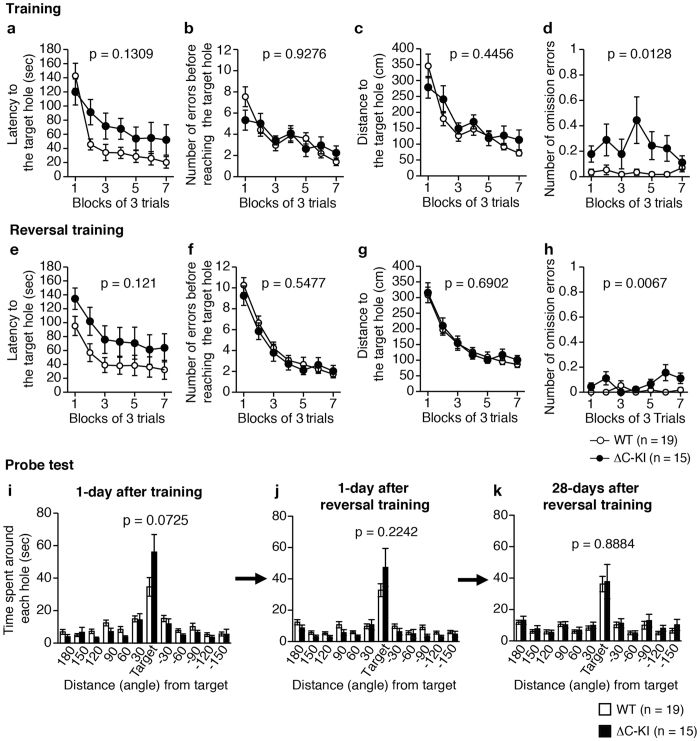
Normal spatial reference memory in ΔC-KI mice. Acquisition training (**a–d**) and reversal training (**e–h**) in the Barnes maze test: Latency to reach the target hole (**a,e**), number of errors before reaching the target hole (**b,f**), distance traveled to reach the target hole (**c,g**), and number of omission errors (**d,h**). (**i–k**) Time spent around each hole in the probe tests at 1 day (**i,j**) or 28 days (**k**) after the last training. Bars represent mean ± SEM. Unpaired t-test (**i–k**) and two-way repeated measures ANOVA (**a–h**) were used to test for statistical significance.

In the training session of the Barnes maze test, there was no significant effect of genotype on the latency to the target hole (Fig. 5a; genotype effect, F_1, 32_ = 2.4, p = 0.1309; genotype × trial interaction, F_6, 192_ = 3.04, p = 0.0074), the number of errors before reaching the target hole (Fig. 5b; genotype effect, F_1, 32_ = 0.01, p = 0.9276; genotype × trial interaction, F_6, 192_ = 1.7, p = 0.1225), or the distance to reach the target hole (Fig. 5c; genotype effect, F_1, 32_ = 0.6, p = 0.4456; genotype × trial interaction, F_6, 192_ = 1.88, p = 0.0868). The number of omission errors (defined by a visit to the target hole without subsequent entry into the target hole) of ΔC-KI mice was increased compared to WT mice (Fig. 5d; genotype effect, F_1, 32_ = 6.95, p = 0.0128; genotype × trial interaction, F_6, 192_ = 2.08, p = 0.0568). Probe tests were performed 1 day after the last training. There was no difference between ΔC-KI and WT mice in the time spent around the target hole (Fig. 5i; t_32_ = 1.86, p = 0.0725).

When the target hole was changed to the opposite side (reversal training), there was no significant effect of genotype on the latency to the target hole (Fig. 5e; genotype effect, F_1, 32_ = 2.54, p = 0.121; genotype × trial interaction, F_6, 192_ = 0.52, p = 0.7914), the number of errors before reaching the target hole (Fig. 5f; genotype effect, F_1, 32_ = 0.37, p = 0.5477; genotype × trial interaction, F_6, 192_ = 0.54, p = 0.7773), or the distance to reach the target hole (Fig. 5g; genotype effect, F_1, 32_ = 0.16, p = 0.6902; genotype × trial interaction, F_6, 192_ = 0.26, p = 0.9563). The number of omission errors of ΔC-KI mice was again increased compared to WT mice (Fig. 5h; genotype effect, F_1, 32_ = 8.42, p = 0.0067; genotype × trial interaction, F_6, 192_ = 3.21, p = 0.005). Probe tests were performed 1 day and 28 days after the last reversal training. There was no difference between ΔC-KI and WT mice in the time spent around the target hole (Fig. 5j,k; genotype effect, t_32_ = 1.24, p = 0.2242 and t_32_ = 0.14, p = 0.8884, respectively), indicating that the acquisition, retention and flexibility of spatial memory are normal in ΔC-KI mice.

### Contextual and cued fear memory in ΔC-KI mice were largely normal

**Figure 6:**
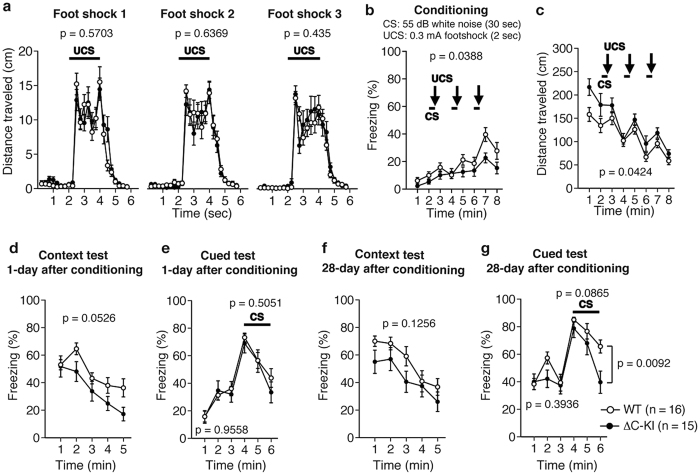
Contextual and cued fear memory in ΔC-KI mice are largely normal. (**a**) Distance traveled during and after each foot shock in the conditioning phase. (**b**) Percentage of freezing response on the conditioning. (**c**) Distance traveled on the conditioning. (**d,e**) Percentages of freezing response on the context (**d**) and cued (**e**) tests at 1 day after conditioning. (**f,g**) Percentages of freezing response on the context (**f**) and cued (**g**) tests at 28 day after conditioning. Two-way repeated measures ANOVA was used to test for statistical significance. Bars represent means ± SEM.

To assess associative fear learning and memory in ΔC-KI mice, we performed the contextual and cued fear conditioning tests. Mice were placed in a chamber and given a conditioned stimulus (CS) of 55 dB white noise, and an unconditioned stimulus (UCS) of foot shock (0.3 mA × 2 sec), three times (conditioning). Responses to foot shocks were not distinguishable between ΔC-KI and WT mice (Fig. 6a; F_1, 29_ = 0.33, p = 0.5703; F_1, 29_ = 0.23, p = 0.6369; F_1, 29_ = 0.63, p = 0.435, for the first, second, and third foot shock, respectively) while the percentage of freezing time in ΔC-KI mice was lower than that in WT mice (Fig. 6b; genotype effect, F_1, 29_ = 4.69, p = 0.0388; genotype × trial interaction, F_7, 203_ = 1.63, p = 0.1276). It is possible that this result was due to hyperactivity in the ΔC-KI mice, given their greater total distance traveled in this test compared to WT mice (Fig. 6c; genotype effect, F_1, 29_ = 4.51, p = 0.0424; genotype × trial interaction, F_7, 203_ = 1.32, p = 0.243). On the following day, the mice were returned to the conditioning chamber and allowed to explore the chamber without either the CS or UCS (context test). There was no difference between ΔC-KI and WT mice in the percentage of freezing time, indicating that contextual fear memory in ΔC-KI mice is normal (Fig. 6d; genotype effect, F_1, 29_ = 4.09, p = 0.0526; genotype × trial interaction, F_4, 116_ = 1.2, p = 0.3137). The cued test was conducted after the context test. Mice were placed in another chamber with different properties from the conditioning chamber. Regardless of presence of the CS, there was no difference between ΔC-KI and WT mice in the percentage of freezing time (Fig. 6e; pre-CS, F_1, 29_ = 0, p = 0.9558; during CS, F_1, 29_ = 0.46, p = 0.5051). These results suggest that cued fear memory in ΔC-KI mice is normal. In the context test performed at 28 days after the conditioning, there was no significant difference in the percentage of freezing time, indicating that remote contextual fear memory in ΔC-KI mice is normal (Fig. 6f; F_1, 29_ = 2.49, p = 0.1256). In the cued test performed at 28 days after the conditioning, there was no significant difference in the percentage of freezing time during pre-CS and CS but ΔC-KI mice showed a reduced percentage of freezing time only during the last 1 min of CS (Fig. 6g; pre-CS, F_1, 29_ = 0.75, p = 0.3936; during CS, F_21, 29_ = 3.15, p = 0.0865; last 1 min of CS, F_1, 29_ = 7.778, p = 0.0092).

### Working memory was impaired but reference memory was normal in ΔC-KI mice

**Figure 7:**
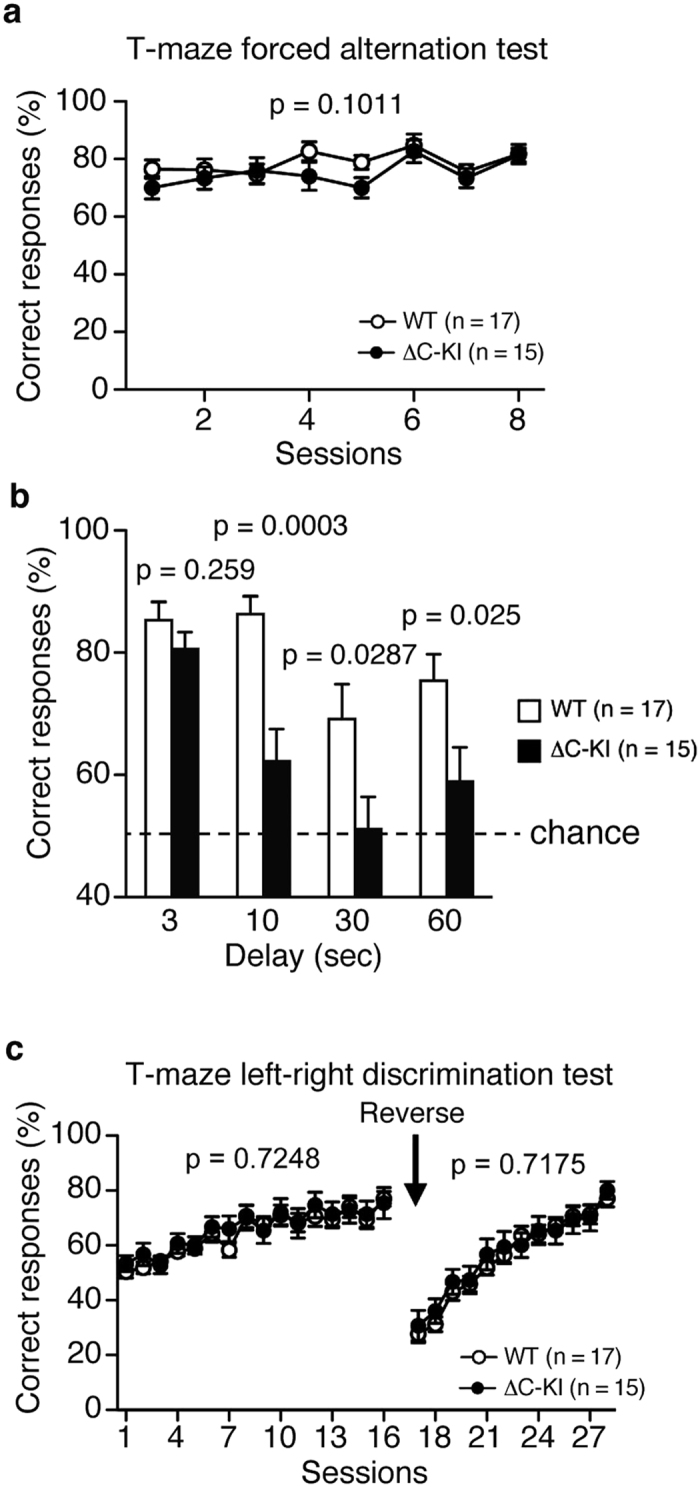
Impaired working memory in ΔC-KI mice. (**a**) Percentage of the correct responses in the training sessions of T-maze forced alternation test. There was no significant difference between the genotypes. (**b**) Percentage of the correct responses in T-maze forced alternation test with delays between forced and free choice runs. (**c**) Percentage of the correct responses in the T-maze left-right discrimination test. Bars represent mean ± SEM. Two-way repeated measures ANOVA (**a,c**) and unpaired t-test (**b**) were used to test for statistical significance.

To assess working memory in ΔC-KI mice, we performed a forced alternation test using a food reward in a T-maze^24^. We weighed mice daily and restricted food throughout the experimental period, feeding them to maintain 80–85% of their free-feeding body weight. During this period mice were given daily training sessions. In the forced alternation test, mice were forced to enter one arm that was baited (forced choice run). In the next run (3 seconds later), mice were allowed to choose one of the arms (free choice run) and were required to enter the arm opposite to the previously baited arm to obtain a food reward. If the mouse remembered the previous run, it would go to the opposite arm. This is considered a correct response. During the training sessions (3 seconds delay), there was no difference between ΔC-KI and WT mice in the percentage of correct responses (Fig. 7a; F_1, 30_ = 2.86, p = 0.1011; genotype × session interaction, F_7, 210_ = 0.62, p = 0.7427). The test was then conducted using 10, 30, or 60-second delays between the forced and free choice runs. In each delay time greater than 3 seconds, ΔC-KI mice exhibited a lower percentage of correct responses than WT mice (Fig. 7b; t_30_ = 1.15, p = 0.259, t_30_ = 4.11, p = 0.0003, t_30_ = 2.3, p = 0.0287, and t_30_ = 2.36, p = 0.025, for 3, 10, 30, 60 seconds, respectively), suggesting that the working memory in ΔC-KI mice is impaired.

To investigate the reference memory in ΔC-KI mice, we performed a left-right discrimination test in T-maze^25^. In this test, the arm where bait was put was fixed, and mice were given free choice runs. There was no difference in the percentage of correct responses between genotypes (Fig. 7c; F_1, 30_ = 0.13, p = 0.7248). Even when the opposing arm was baited instead, there was no difference in the percentage of correct responses (Fig. 7c; F_1, 30_ = 0.13, p = 0.7175). These results indicated that the acquisition, retention and flexibility of reference memory in ΔC-KI mice are normal, consistent with the results of the Barns maze test described in the “spatial reference memory” section (Fig. 5).

## Discussion

In this study, we identified several key anatomical and behavioral features related to Reelin signaling using ΔC-KI mice. First, in ΔC-KI mice, the structural abnormalities of the cerebral cortex and hippocampus in neonates persisted into adulthood, as did the attenuated Reelin signaling. Second, ΔC-KI mice were hyperactive. Third, ΔC-KI mice exhibited reduced anxiety-like behavior and reduced sociability. Fourth, the working memory in ΔC-KI mice was partially impaired. Fifth, spatial reference memory, PPI, depression-like behavior, and fear memory in ΔC-KI mice were essentially intact. These results provide novel insights into Reelin functions in the developing and adult brain, and their potential association with neuropsychiatric disorders. In particular, these data indicated that the loss of Reelin CTR and/or reduced Reelin signaling could be the direct cause of some symptoms observed in schizophrenia, bipolar disorder, or ASD.

A number of studies have suggested some form of a relationship between hypoactivity of Reelin and neuropsychiatric disorders^3^,^10^. For example, genomic studies reproducibly indicated that variations of the *Reelin* gene are associated with schizophrenia^26^,^27^,^28^,^29^, bipolar disorder^29^,^30^, and ASD^31^,^32^. Single nucleotide polymorphisms in the Reelin receptor ApoER2, as well as decreases in its mRNA, are also significant risk factors for schizophrenia and bipolar disorder^33^,^34^. In post-mortem brains of patients with schizophrenia and bipolar disorder, DNA methylation and hydroxymethylation at the *Reelin* promoters are altered^35^ and Reelin protein in the prefrontal area is decreased^36^,^37^,^38^. It is unknown whether the abnormality in the Reelin pathway observed in these studies affected brain development required for normal brain functions or it directly induced the adult brain malfunctions. On the other hand, anti-schizophrenic drugs augment Reelin expression^39^,^40^,^41^ and injection of Reelin protein into the ventricle of HRM restores normal hippocampal synaptic function, associative learning and memory, and PPI^42^. Reelin injection also counteracts phencyclidine-induced deficits in cognitive and sensorimotor gating in WT mice^43^. Reelin overexpression in the mouse forebrain prevents the manifestation of behavioral phenotypes related to schizophrenia and bipolar disorder^16^. Finally, Reelin dynamically induces transcriptional changes in primary cortical neurons and many Reelin target genes are positively associated with genes implicated in neuropsychiatric disorders and in neuronal plasticity^9^. All of these observations point to the importance of correct Reelin signaling in the adult brain and suggest that hypoactivity of Reelin causes deterioration in neuropsychiatric symptoms.

However, the function of Reelin in the adult brain is still incompletely understood, and its significance in neuropsychiatric disorders is far from proven. In particular, the consequences of Reelin hypoactivity in adult mice have been investigated solely using HRM, with one recent exception^21^, and there are some discrepancies among the published research. For example, some groups^11^,^17^,^44^,^45^ reported that PPI, a relatively well-validated endophenotype for schizophrenia, was reduced in HRM. However, others^18^,^19^,^21^ reported the opposite. Schroeder and colleagues^20^ even argued that HRM are less susceptible to corticosterone-induced PPI deficits than WT mice. Moreover, HRM are typically maintained on background strains such as B6C3Fea/a for historical reasons. This makes comparison with some studies, in which C57BL/6 strain was used, difficult. Our ΔC-KI mice are the only knock-in mice to date in which the function of *Reelin* gene is partially impaired and they reflect some morphological abnormalities observed in patients with certain neuropsychiatric disorders^22^. Therefore, the present study can provide important information regarding the relationship between Reelin activity and higher brain functions. It should be emphasized that Reelin activity is diminished from developmental stages thorough adulthood in our ΔC-KI mice, as in HRM, and thus future studies are needed to strictly clarify whether their behavioral phonotypes due to developmental/anatomical defects or to adult synaptic defects.

Among the behavioral phenotypes of ΔC-KI mice, hyperactivity is the most striking. We assume that hyperactivity is the main cause of some of the other behavioral phenotypes of ΔC-KI mice, including lighter body weight (Fig. 2a), reduced wire hang latency (Fig. 2e), and increased rotarod latency (Fig. 2f). Hyperactivity has been observed in HRM^46^, VLDLR KO mice^47^, Dab1 *scrambler* mutant mice^48^, and adult-specific^49^ or dorsal forebrain-specific^50^ Dab1 KO mice, but the degree of hyperactivity of ΔC-KI mice is much higher. Therefore, it is conceivable that reduced Reelin signaling generally causes hyperactivity in mice but the CTR-dependent function is absolutely required for controlling locomotor activity. Interestingly, Reelin expression in rats that received prenatal restraint stress was decreased and they too were shown to be hyperactive^14^. Altogether, these results suggest that Reelin signaling in both the developing and adult brain play a role in suppressing hyperactivity, with developmental role being more important.

Reduced anxiety-like behavior is also a characteristic phenotype of ΔC-KI mice. Such reductions have been reported in HRM^12^,^51^ (but not in one study^17^), adult-specific Reelin KO mice^21^, Dab1 *scrambler* mutant mice^48^, and dorsal forebrain-specific Dab1 KO mice^50^, supporting the idea that Reelin-Dab1 signaling in the adult brain helps maintain a normal level of anxiety-like behavior. One report against this scenario is that an adult forebrain excitatory neuron-specific Dab1 KO had no effect on anxiety-like behavior^52^. This apparent contradiction may be reconciled if Dab1 is required in the developing stages and Dab1-independent Reelin function is required in adult brain. In addition, Dab1 has a role other than as a downstream signal for Reelin^53^, and Reelin has a Dab1-independent effect as well^54^. We also showed that the CTR of Reelin binds to a specific molecule(s) on neuronal cell membrane^22^. Therefore, many problems currently muddy the picture, and need to be solved before our understanding of Reelin’s function in regulating anxiety-like behavior is clear.

The social behavior of ΔC-KI mice was found to be slightly abnormal as well. To the best of our knowledge, sociability has never been tested rigorously using HRM, but published data do provide evidence of some abnormalities that could be interpreted as reminiscent of symptoms of ASD^10^,^12^. Such ASD-like symptoms would include the stereotypy we identified in the open field test (Fig. 3c)^50^. As slight reduction of Reelin signaling is considered a great risk in ASD^32^,^55^, the ΔC-KI mice should be useful for analyzing the contribution of Reelin hypoactivity to the onset of ASD. They may even make a good model of ASD and it would be interesting to test whether proposed treatments against ASD, such as oxytocin administration, improve the sociability of ΔC-KI mice.

Working memory is a system for temporarily storing and managing the information required to carry out complex cognitive tasks, and its deficit is a well-validated cognitive symptom of neuropsychiatric disorders. In this study, we found that the working memory in ΔC-KI mice is impaired (Fig. 7b). Interestingly, working memory in HRM is reported to be normal^56^,^57^,^58^, while it was abnormal in adult forebrain excitatory neuron-specific Dab1 KO mice^50^. It was thus suggested that 50% reduction of total Reelin amount has negligible effect for normal working memory formation but CTR-dependent, reinforced Dab1 phosphorylation^22^ in the adult brain is required for it. Working memory was not investigated in adult-specific Reelin KO mice^21^. In human, microsatellites of *Reelin* are highly associated with working memory^27^,^59^, and there are ample *in vitro* studies indicating that Reelin plays critical roles in synaptic formation and plasticity^3^. Therefore, it is very likely that the full activity of Reelin, exerted by Reelin with intact CTR, is required for normal working memory function and that its deficit can lead to onset or deterioration of neuropsychiatric disorders such as schizophrenia.

In conclusion, our study shows that ΔC-KI mice show multiple behavioral phenotypes that are reminiscent of some behavioral symptoms in various neuropsychiatric disorders including schizophrenia, bipolar disorder, and ASD. Among them, hyperactivity and ASD-like symptoms are likely to derive from developmental or anatomical defects while reduced anxiety-like behavior is from reduced Reelin functions in adult brain, since only the latter was observed in adult-specific Reelin KO mice^21^. It would be safe to say that our ΔC-KI mice mimic human patients who have genetic deficits in *Reelin* or its downstream genes, or who have structural abnormalities in their brain.

Future studies are required to clarify the neuronal network or system(s) defective in ΔC-KI mice, and the molecular mechanisms underlying them.

## Materials and Methods

### Animals

All of the experimental protocols were approved by the Animal Care and Use Committee of Nagoya City University and by Fujita Health University, and were performed according to the guidelines of the National Institutes of Health of Japan. Generation of ΔC-KI mice was previously described^22^. All mice were backcrossed to the C57BL/6N mice for at least ten generations. WT and ΔC-KI mice were generated by intercrossing with heterozygous male and female mice. Mice older than 11-weeks old were used for behavioral tests. Mice were housed two to four per cage (one to three for each genotype) in a room with a 12 h light/dark cycle (lights on at 7:00 am), with access to food and water ad libitum except for the period during which the T-maze test was performed. Room temperature was kept at 23 ± 2 °C. Behavioral tests were performed between 9:00 a.m. and 6:00 p.m. Before all behavioral tests, mice were left in the testing room for at least 30 min to allow acclimation. After the test, the testing apparatus was cleaned with diluted sodium hypochlorite solution to prevent a bias due to olfactory cues.

### Immunohistochemistry

Mice were transcardially perfused with 4% paraformaldehyde in PBS. Fixed brains were immersed sequentially in 20% and 30% sucrose in PBS for cryoprotection, then embedded in OCT Compound (Sakura Finetek, Tokyo, Japan), and frozen on dry ice. The frozen brains were coronally sectioned at 14 μm using a cryostat (CM1850; Leica Microsystems). Immunohistochemistry with anti-NeuN antibody (1:500; EMD Millipore, Billerica, MA) was performed as described previously^22^.

### Western blotting

Dissected cerebral cortices and hippocampi were homogenized in lysis buffer (20 mM Tris-HCl, pH 7.5, 150 mM NaCl, 5 mM EDTA, 1% Triton X-100, 0.1% H_2_O_2_, and 5 mM Na_3_VO_4_). Insoluble debris was removed by centrifugation (10 min, 17,800 g), and supernatants were diluted with 4 x SDS-PAGE loading buffer (0.25 M Tris-HCl, pH 6.8, 40% (w/v) glycerol, 8% SDS, 20% (v/v) 2-ME, 0.2% BPB). Samples were separated by SDS-PAGE (5% and 8% separating gels for Reelin and Dab1, respectively). Western blotting was performed as described previously^22^. Anti-Reelin G10 (1:1,000) antibody was purchased from Millipore. Anti-Dab1 monoclonal antibodies (1:1,000) were described previously^60^. Anti-α-tubulin (1:10,000) was purchased from Wako Pure Chemicals (Osaka, Japan). Images were captured using a LAS4000 system (Fuji, Tokyo, Japan) and were quantified with ImageJ software (version 1.48v, National Institutes of Health, MD) as described previously^23^.

### Comprehensive behavioral test battery

Most of the behavioral tests were performed as previously described^25^, unless otherwise noted.

### The Barnes maze test

For the first three days, two trials per day were conducted. For the fourth day onward, three trials per day were conducted. Approximately 24 h after the last training session, a probe test (3 min) was conducted without the escape box, to assess memory based on distal environmental room cues. After probe test, three trails were conducted, and then the target hole was changed to the opposite side. Three reversal trials per day were conducted subsequently. Approximately 24 h after the last reversal training session, a probe test (3 min) was conducted. Another probe test was conducted 28 days after the last reversal training session to evaluate memory retention.

### T-maze test

Forced alternation and left-right discrimination tests using food reward were conducted as previously described^24^. One week before the pre-training, mice were deprived of food until their body weight was reduced to 80–85% of their initial weight. Mice were then kept on a maintenance diet throughout the T-maze tests. On the following four days after the habituation session, mice were subjected to pre-training, during which they were allowed to consume the food reward in each arm of the T-maze apparatus. On the day after the pre-training sessions, mice were subjected to a forced alternation task for 8 days (one session consisting of 10 trials per day; cut-off time, 50 min). Thereafter, for 3 days, mice were subjected to the forced alternation test with a delay (3, 10, 30, or 60 s), which was interposed/placed/inserted between the forced choice run and the free choice run.

### Home cage social interaction test

The social interaction monitoring system comprised a home cage and a filtered cage top with an infrared video camera (25 × 15 × 23.5 cm, inside dimensions). Two mice of the same genotype that had been housed separately were placed together in the home cage. Output from the video camera was fed into a computer. Images from each cage were captured at a rate of 1 frame per sec. Social interaction was measured by counting the number of particles detected in each frame. Two particles indicated that the mice were not in contact with each other, and one particle indicated contact between the two mice. We also measured the activity level of the mice by quantifying the number of pixels that changed between each pair of successive frames. The mean number (±SD) of pixels of adult male C57BL/6J mice (n = 8; body weights, 26–43 g) in an image was 415 ± 69. When two mice move, the maximum number of pixels can be more than 1,500 per sec. We defined as immobile the state when the number of pixels was less than 1,500 during a 1-min period, and evaluated the mean number of particles during immobile and active periods. Analysis was performed automatically using ImageHA software developed by T. Miyakawa.

### Statistical analyses

All quantitative data were expressed as mean ± SEM. Western blot data were analyzed using unpaired t-test in GraphPad Prism6 (GraphPad Software, CA, USA). Behavioral data were analyzed using unpaired t-test, paired t-test, or two-way repeated measures ANOVAs in SAS University Edition (SAS institute, NC, USA). P-values < 0.05 were considered to denote significance.

## Additional Information

**How to cite this article**: Sakai, K. *et al*. Mice that lack the C-terminal region of Reelin exhibit behavioral abnormalities related to neuropsychiatric disorders. *Sci. Rep.* **6**, 28636; doi: 10.1038/srep28636 (2016).

## Supplementary Material

Supplementary Information
