# Supplementary material for: Mice that lack the C-terminal region of Reelin exhibit behavioral abnormalities related to neuropsychiatric disorders
Source: Sci Rep. 2016 Jun 27;6:28636. doi: 10.1038/srep28636 (PMC4921851; doi:10.1038/srep28636)
Supplement: Supplementary Information [file srep28636-s1.pdf]

Supplemental Figures

Mice that lack the C-terminal region of Reelin exhibit behavioral abnormalities related to neuropsychiatric disorders

Kaori Sakai, Hirotaka Shoji, Takao Kohno, Tsuyoshi Miyakawa, and Mitsuharu Hattori

Supplementary Figure S1: Normal depression-like behavior of ΔC-KI mice.

(a) Immobility of ΔC-KI and WT mice on day 1 and 2 in the Porsolt forced swim test. (b) Immobility of ΔC-KI and WT mice in tail suspension test. Bars represent mean ± SEM. Two-way repeated measures ANOVA was used to test for statistical significance.

Supplementary Figure S2: Decreased social interaction during inactive state in ΔC-KI mice.

Mean number of particles was presented in different activity levels. Bars represent mean ± SEM. Two-way repeated measures ANOVA was used to test for statistical significance.

Supplementary Figure S3: Normal acoustic startle response and prepulse inhibition (PPI) of ΔC-KI mice.

(a) Amplitude of the startle response. (b) Percentage of PPI. Bars represent mean ± SEM. Unpaired t-test was used to test for statistical significance.

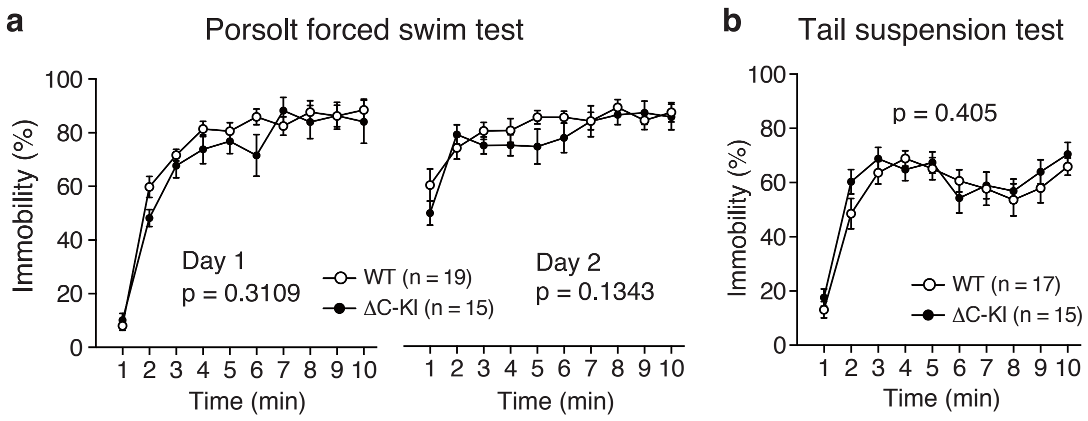

Sakai et al. Supplemetary Figure 1

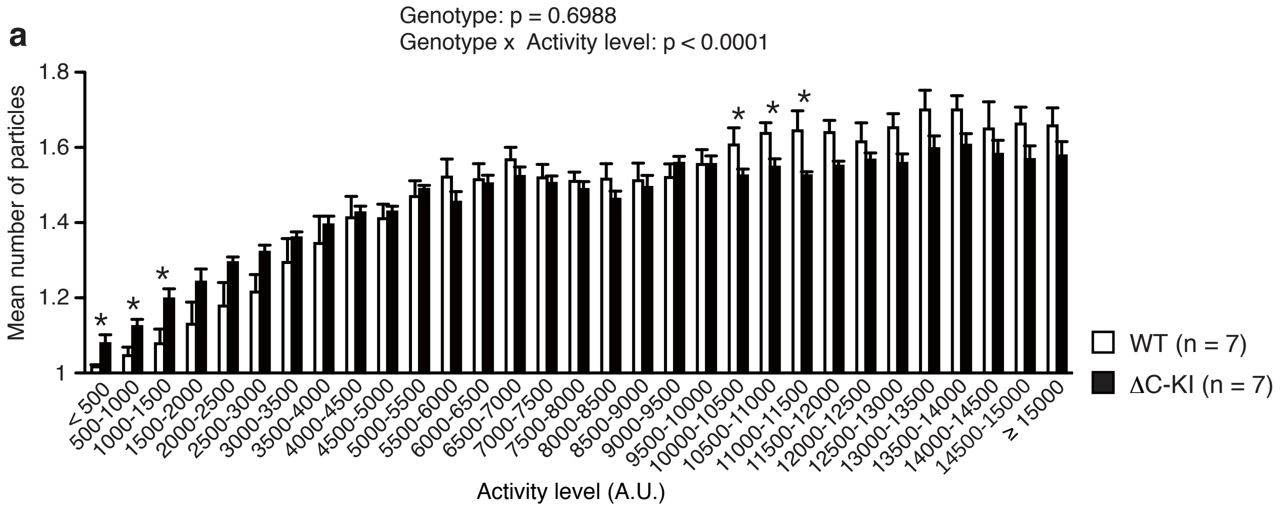

Sakai et al. Supplementary Figure 2

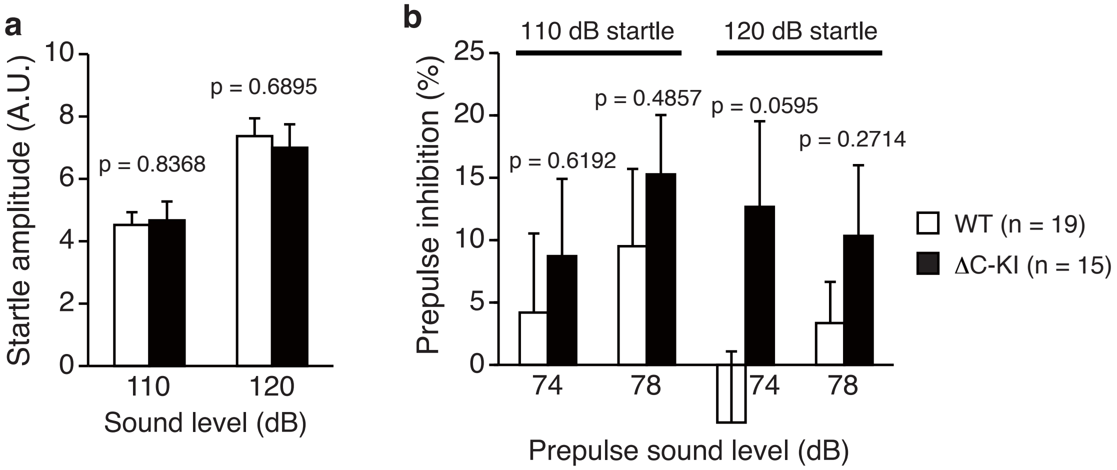

Sakai et al. Supplementary Figure 3
